# Chaotic Mapping Lion Optimization Algorithm-Based Node Localization Approach for Wireless Sensor Networks

**DOI:** 10.3390/s23218699

**Published:** 2023-10-25

**Authors:** Abdelwahed Motwakel, Aisha Hassan Abdalla Hashim, Hayam Alamro, Hamed Alqahtani, Faiz Abdullah Alotaibi, Ahmed Sayed

**Affiliations:** 1 Department of Electrical and Computer Engineering, International Islamic University Malaysia, Kuala Lumpur 53100, Malaysia; 2 Department of Management Information Systems, College of Business Administration in Hawtat Bani Tamim, Prince Sattam bint Abdulaziz University, Al-Kharj 16278, Saudi Arabia; 3 Department of Information Systems, College of Computer and Information Sciences, Princess Nourah bint Abdulrahman University, P.O. Box 84428, Riyadh 11671, Saudi Arabia; 4 Department of Information Systems, College of Computer Science, Center of Artificial Intelligence, Unit of Cybersecurity, King Khalid University, Abha 61421, Saudi Arabia; 5 Department of Information Science, College of Arts, King Saud University, P.O. Box 28095, Riyadh 11437, Saudi Arabia; 6 Research Center, Future University in Egypt, New Cairo 11835, Egypt

**Keywords:** anchor nodes, metaheuristic optimization algorithm, node localization, tent chaotic mapping, wireless sensor networks

## Abstract

Wireless Sensor Networks (WSNs) contain several small, autonomous sensor nodes (SNs) able to process, transfer, and wirelessly sense data. These networks find applications in various domains like environmental monitoring, industrial automation, healthcare, and surveillance. Node Localization (NL) is a major problem in WSNs, aiming to define the geographical positions of sensors correctly. Accurate localization is essential for distinct WSN applications comprising target tracking, environmental monitoring, and data routing. Therefore, this paper develops a Chaotic Mapping Lion Optimization Algorithm-based Node Localization Approach (CMLOA-NLA) for WSNs. The purpose of the CMLOA-NLA algorithm is to define the localization of unknown nodes based on the anchor nodes (ANs) as a reference point. In addition, the CMLOA is mainly derived from the combination of the tent chaotic mapping concept into the standard LOA, which tends to improve the convergence speed and precision of NL. With extensive simulations and comparison results with recent localization approaches, the effectual performance of the CMLOA-NLA technique is illustrated. The experimental outcomes demonstrate considerable improvement in terms of accuracy as well as efficiency. Furthermore, the CMLOA-NLA technique was demonstrated to be highly robust against localization error and transmission range with a minimum average localization error of 2.09%.

## 1. Introduction

Wireless sensor networks (WSNs) comprise a variety of mobile or static sensors in multi-hop and self-organizing ways, targeted at transferring the data processed and identified by the sensor nodes (SNs) from the coverage zone of the network to users [1]. WSNs incorporate MEMS and sensor technology with network transmission technology and are extensively employed in these domains, namely intelligent transportation, environmental safety, agriculture, military, and other domains [2]. They can be the focus of international competition due to the considerations of the academic community and various industries [3]. Since the location data of nodes perform a main part in the processing method of WSNs [1], localization is an essential fundamental technology. While the existing GPS localization system can exactly locate the target, it has been challenging to utilize satellite positioning data for correctly positioning the target in any specific location [4]. Hence, it requires a highly significant and complex effort to analyze the accurate localization technique for WSNs [5].

Numerous non-GPS-based localization techniques have been exploited, which can be classified into two categories, namely range-free and range-based methods [6]. A range-based localization approach employs point-to-point distance evaluation or angle-based assessment amongst SNs [7]. In this, a location can be estimated via the trilateration of anchor nodes (ANs) (whose locations can be recognized) [8]. Range-free localization approaches do not need range data among ANs and target nodes (TNs) but rely on topological data [9]. The range-based method provides a higher accuracy as compared to range-free localization techniques; however, it is not very cost effective [10]. The range-based localization of SNs is achieved using two stages, namely ranging and position estimation stages. In the primary stage, all TNs estimate their distance from the ANs utilizing the intensity of the receiving signal or the signal transit time. Accurate distance evaluation is impossible due to noise [11]. In the secondary stage, the location of the SNs is detected by the data acquired from the ranging stage. This can be performed through both geometric methods and optimization methods. 

Previously, several challenging techniques have been employed to face the difficulty of WSN node localization (NL) [12]. Some bio-inspired methods for localization are presented that can also be analyzed in this paper, namely a bat optimization algorithm (BA), swarm-based algorithms, evolutionary algorithms, and others stimulated by animal social activity [13]. It is a new technique for emerging innovative computing algorithms that depend on natural development [14]. Various models have been exploited in the development of these techniques like mentioning the responses and behaviors of animals in the wild and making a paradigm to represent their behaviors. Alternatively, bio-inspired optimization approaches could be analyzed to obtain parameter settings for enhancing the performance of this developed approach [15].

This paper develops a Chaotic Mapping Lion Optimization Algorithm-based Node Localization Approach (CMLOA-NLA) for WSNs. The purpose of the CMLOA-NLA system is to define the localization of unknown nodes (UNs) based on the ANs as a reference point. In addition, the CMLOA is mainly derived from the integration of the tent chaotic mapping concept into the traditional LOA, which tends to improve the convergence speed and precision of node localization. With extensive simulations and comparison results with recent localization approaches, the effectual performance of the CMLOA-NLA technique is illustrated. In short, the key contributions of the paper are given as follows.

- Design a new CMLOA-NLA technique to effectually determine the location of the nodes based on ANs, which enhances the localization by leveraging chaotic mapping concepts, leading to faster convergence and more precise results.
- CMLOA-NLA demonstrates robustness against localization errors and variations in transmission range, making it suitable for real-world WSN deployments where environmental factors can introduce uncertainty.

The rest of the paper is organized as follows. Section 2 provides the related works and Section 3 offers the proposed model. Then, Section 4 gives the result analysis and Section 5 concludes the paper.

## 2. Related Works

Agarwal et al. [16] considered the development of an Intelligent Aquila Optimizer Algorithm-Based NL Scheme (IAOAB-NLS) for WSNs. This introduced approach creates the application of ANs for detecting a suitable NL. Additionally, the IAOAB-NLS algorithm could be inspired by Aquila behavior. The IAOAB-NLS technique can have the capability of achieving suitable coordinated points of the nodes in the network. In [17], an efficient metaheuristic-based Group Teaching Optimizer Algorithm was developed for the NL (GTOA-NL) method for WSNs. To guarantee the effectual NL performance of this introduced GTOA-NL approach, a wide-ranging set of models was implemented to focus on the excellence of the GTOA-NL approach. Cheng et al. [18] developed a Modified Archimedes Optimizer Algorithm (MAOA) and DV-Hop termed as MAOADV-Hop. Primarily, PSO and tent chaotic mapping techniques have been presented in AOAs. Secondarily, the MAOA was exploited to exchange the least square (LS) part of the DV-Hop localized method.

In [19], an NL method for WSNs that depends on virtual partition and distance correction (VPDC) was developed. Primarily, the distance of every hop on a shorter transmission path between the UN and beacon node is considered through the VP approach; next, the shorter communication path length was acquired by including every hop distance; at last, the distance among nodes was accomplished using the optimum path search method and the DC procedure. Soundararajan et al. [20] introduced a metaheuristic optimization-based NL and multi-hop routing protocol with MS (MONL-MRPMS) for WSNs. This method targets to attain energy efficiency with accurate NL effectiveness and includes an efficacious Coyote Optimizer Algorithm (COA) for NL (COA-NL) in WSNs. Furthermore, a seagull optimization-based multi-hop routing (SGO-MHR) model was developed for optimally choosing routes for inter-cluster transmission. In [21], an innovative iterative localization approach termed CVX-DV-Hop was developed in this communication. Firstly, it achieves matrix transformation for reformulating the original optimizer issue into one with nonconvex and convex constraints. Secondly, first-order Taylor expansions have been utilized to compress the nonconvex constraints into linear inequality limits. At last, a successive convex approximation technique was developed for iteratively solving the optimization difficulties.

Yu et al. [22] recommended a quantum annealing BA (QABA) approach for NL in WSNs. QABA integrates quantum development and an annealing approach to the model of the BA for enhancing global and local search capability and lastly, converging to better-optimized values. In [23], an opposition-based learning and parallel strategy AGTO (OPGTO) technique was developed. This study develops communication schemes among clusters for various kinds of optimizer issues. Eventually, the OPGTO technique is implemented for the 3D localization of WSNs in real time.

An important research gap in the domain of WSN node localization is the pressing need for improving metaheuristics. However, considerable efforts are dedicated to emerging localization systems as the existing approaches frequently struggle to deliver the needed accuracy, energy efficacy, and scalability required by real-world WSN deployments. Enhanced metaheuristics are vital for addressing these problems by optimizing localization systems for handling noisy sensor data, adjusting to dynamic environmental conditions, minimizing energy consumption, and ensuring robustness in the face of node failures or adversarial attacks. Bridging this gap in metaheuristic research could advance the field of WSN node localization to enable the extensive and reliable execution of WSNs in various applications.

## 3. The Proposed Model

**Figure 1:**
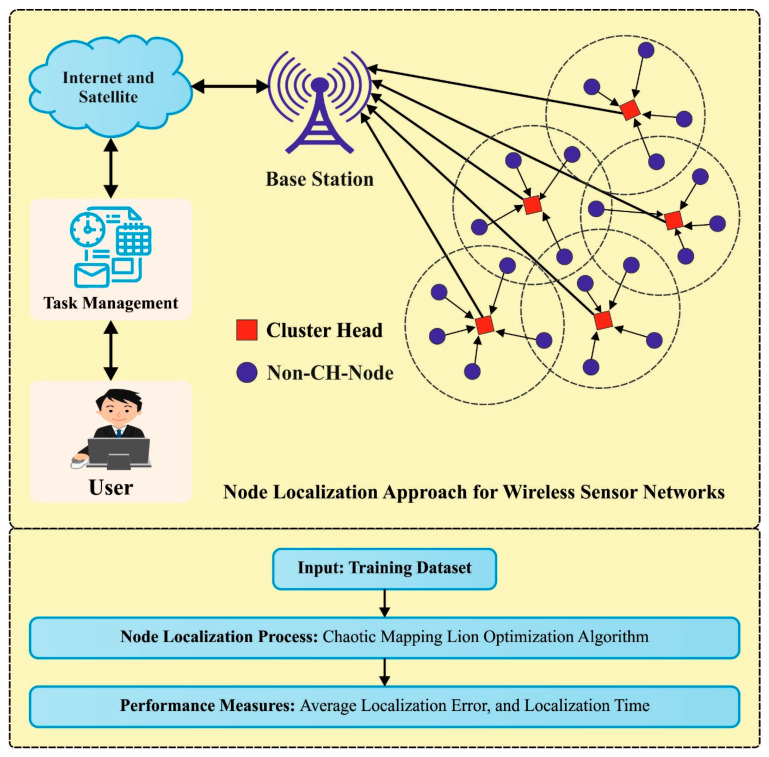
The overall process of the CMLOA-NLA method.

In this manuscript, a novel localization approach using a metaheuristic algorithm, named CMLOA-NLA, has been developed for WSNs. The purpose of the CMLOA-NLA system is to define the localization of the UNs based on the ANs as a reference point. Figure 1 illustrates the overall procedure of the CMLOA-NLA algorithm.

### 3.1. Problem Formulation

UNs and ANs are important for the NL problem in WSNs [24]. The energy consumption and cost of ANs are generally far greater than the typical SN. The localization technique is to optimize and find the location of the UN via ANs with known positions. The explanation of the NL problem is given in the following: (1) there are m anchors with known locations and n UNs with unknown positions; (2) consider that the ANs and UNs of the sensor network can be distributed on an sL×L 2D plane and the nodes are uniformly and randomly distributed; (3) the measured distance error conforms to uniform distribution and the measured distance is $d_{ij}^{\prime }$, as given in Equation (1):

(1) $$d_{ij}^{\prime }=d_{ij}\cdot \left(1+\tau \cdot \varepsilon \right),\;$$

In Equation (1), $d_{ij}$ shows the real distance from the *i*th to *j*th nodes, the distribution of ε is σ(0,1), and τ refers to the error feature.

The classical DV-Hop method without range-based positioning comprises three stages: computing the UN coordinate location, computing the minimum number of hops (MNH) among anchors, and computing the evaluated distance from ANs to UNs. 

The basic steps of the DV-Hop method are discussed below.

Step 1: the MNH can be computed by the flooding method, where the anchor broadcasts data to all the nodes of the WSN. Later the MNH between the UNs and ANs is computed, along with the MNH between the ANs themselves.

Step 2: the mean distance per hop $Hop_{i}$ of all the ANs and the evaluated distance $d_{u,\;i}$ is measured by Equation (2):

(2) $$Hop_{i}=\frac{\Sigma_{i\neq j}d_{i,j}}{\Sigma_{i\neq j}h_{i,j}}=\frac{\Sigma_{i\neq j}\sqrt{{\left(x_{i}-x_{j}\right)}^{2}+{\left(y_{i}-y_{j}\right)}^{2}}}{\Sigma_{i\neq j}h_{i,j}}\;$$

where $\left(x_{i},y_{i}\right)$ shows the location coordinate of *i*th ANs, $\left(x_{j},x_{j}\right)$ indicates the location coordinate of *j*th ANs, $h_{i,j}$ shows the MNH from *i*th AN to *j*th nodes, and evaluated distance $d_{u,\;i}$ from *i*th AN to u UN is expressed by Equation (3):

(3) $$d_{u,i}=Hop_{i}\times h_{u,i}$$

where $h_{u,\;i}$ signifies the MNH from UN u to *i*th AN.

Step 3: the fitness function (FF) of the NL optimizer technique can be evaluated by Equation (4):

(4) $$f\left(x_{u}\right)=\overset{m}{\underset{i=1}{\sum }}\sqrt{{\left(x_{u}-x_{i}\right)}^{2}+{\left(y_{u}-y_{i}\right)}^{2}}-d_{u,i}$$

Here $\left(x_{u},\;y_{u}\right)$ indicates the evaluated location coordinate of uth UNs, $\left(x_{j},y_{j}\right)$ represents the location coordinate of *i*th ANs, $d_{u,i}$ indicates the evaluated distance from *u*th UNs to *i*th Ans, and m indicates the number of ANs.

### 3.2. Modeling of CMLOA

LOA is a metaheuristic approach where a set of random solutions called lions establish a population initialization [25]. N solution constitutes the population where all the solutions contain α and β features that should be enhanced. This can be elaborated in the following:

(5) Solution (Lion)=[α, β]

Some lions in the (N) population procedure are nomads and the residual populations are randomly selected as Prides (P). Amongst nomad lions, S% of individuals are female and the residue is male. The solution is selected as follows, using various integrations of contrast and brightness. The Hunter will continuously improve its fitness, and simultaneously, PREY will attempt to get away from the hunter and attain its novel location as given in Equation (6):

(6) $$PREY^{\prime }=PREY+rand\left(0,1\right)\times PI\times \left(PREY-Hunter\right)$$

In Equation (6), PREY shows the existing location of the prey, Hunter indicates the new location utilized by the hunter to attack the target, and PI shows the % of hunter fitness enhancement, as shown in Equation (7):

(7) $$Hunter^{\prime }=\{\begin{array}{l} rand\left(\left(2\times PREY-Hunter\right),PREY\right),\\ \left(2\times PREY-Hunter\right)<PREY\\ rand\left(PREY,\left(2\times PREY-Hunter\right)\right),\\ \left(2\times PREY-Hunter\right)>PREY \end{array}\;$$

where Hunter represents the existing hunter location and Hunter shows the novel location of the hunter. The upgraded position of the center hunter is shown in Equation (8).

(8) $$Hunter^{\prime }=\{\begin{array}{cc} rand\left(Hunter,\;PREY\right), & Hunter<PREY\\ rand\left(PREY,Hunter\right), & Hunter>PREY \end{array}\;$$

The territory of each pride has its own best solution, i.e., the member which helps to retain the optimum solution for the algorithm. The new position of the female lion is shown in Equation (9):

(9) $$Female\;Lion^{\prime }=Female\;Lion+2\times D\times rand\left(0,1\right)\left\{R1\right\}\;+U\left(-1,1\right)\times tan\left(\;\right)\times D\times \left\{R2\right\}$$

{R1}.{R2}=0

||{R2}||=1

where Female Lion represents the existing location of lions, and D shows the lion location recognized by the selection tournament from the pride territory [26]. The value of {R1} shows the initial position—that is, the prior location of the lions—and heads nearby {R2}. The {R1} and {R2} vectors are perpendicular to one another. Also, the resident male lion roams toward a random location, and once the novel position is superior to the prior one, it instantaneously upgrades the local optimum performance. Figure 2 depicts the flowchart of LOA.

Then, mating can be carried out to generate new offspring. The pre-determined c% of female lions from all of the pride gets crossed over with multiple random resident males. However, the nomad lion mates only with one random male. The two offspring are generated using Equations (10) and (11) after a pair for mating is selected.

(10) $$Offspring_{j}1=\beta \times Female\;Lion_{j}+\overset{\;}{\underset{\;}{\sum }}\frac{1-\beta }{\sum_{i=1}^{NR}S_{i}}\times MaleLion_{j}^{i}\times S_{i}$$

(11) $$Offspring_{j}2=\left(1-\beta \right)\times Female\;Lion_{j}+\overset{\;}{\underset{\;}{\sum }}\frac{\beta }{\sum_{i=1}^{NR}S_{i}}\times MaleLion_{j}^{i}\times S_{i}$$

where j refers to dimension, $S_{i}$ takes the value of 1 if the *i*th male has been applied for crossover and 0 or else, NR shows the resident male counts present from the pride, and β denotes a randomly generated number that follows a standard distribution with a standard deviation of 0.1 and mean value of 0.5. Two randomly produced offspring are selected as female and male. M% of genes are mutated, whereas arbitrary numbers replace them. LOA produces a population of the newest cubs with novel inherited features in the parent by completing the operation. The defense operation is efficiently performed where the mature male aggressively fights with other individuals [26]. The beaten lion with lower fitness is detached from the pride and becomes a nomad, but the high fit lion is taken from the population by becoming the resident male.

In the process of migration, some random females develop to be nomads and could be migrated out of the pride. New and old nomads are arranged by the fitness value and the optimum one is pushed again to the population to fill the position of the eliminated lion. This procedure makes sure that an appropriate amount of diversity is often preserved in the population.

The CMLOA is mainly derived from the integration of the tent chaotic mapping concept into the traditional LOA. The tent chaotic map enhances the population diversity to power the global searching ability of the method [27]. The mathematical formula is depicted in Equation (12):

(12) $$x_{i+1}=\{\begin{array}{l} \frac{\chi_{i}}{a},\;\;\;\;\;\;\;\;\;\;\;\;\;if\;x_{j}<a,\\ \frac{x_{i}}{\left(1-a\right)},\;\;\;if\;x_{i}\geq a, \end{array}i=1,2,\ldots ,\;D,$$

where a denotes the chaos factor, and a=0.7. D refers to the population dimension.

### 3.3. Steps Involved in CMLOA-NLA Technique

The CMLOA-NLA algorithm involves the following steps to localize the sensor in WSNs. N AN and M TN are randomly placed from the device part [28]. All the ANs are spatially localized and helped to identify the residence of other nodes. All the targets and ANs comprise the transmission range R.

(1) The distance between the ANs and TNs is altered and evaluated using preservative Gaussian noise. The TN is used to control distance as ${\hat{d}}_{i}=d_{i}+n_{i}$, where $d_{i}$ signifies the actual distance, viz., calculated amongst the place of beacon $\left(x_{i},y_{i}\right)$ and the place of TN (x,y). It is defined in Equation (13).

(13) $$d_{i^{=}}\sqrt{{\left(x-x_{i}\right)}^{2}+{\left(y-y_{i}\right)}^{2}}.$$

The parameter $n_{j}$ controls the noise that traces the evaluated distance in $d_{i}\pm d_{i}\left(P_{n}/100\right)$, where $P_{n}$ signifies the sound connection within the predicted distance.

(2) The chosen node is called a localizable node once it takes three ANs in the communication radius (CR) of TN.

(3) For the localized node, the CMLOA-NLA method was separately executed to identify the place of TN. The CMLOA-NLA technique is implemented by the centroid of AN inside a CR, as provided in Equation (14).

(14) $$\left(x_{c},y_{c}\right)=\left(\frac{1}{N}\overset{N}{\underset{i=1}{\sum }}x_{i},\frac{1}{N}\overset{N}{\underset{i=1}{\sum }}y_{i}\right)$$

Now N shows the entire AN counts in the communication range of restricting TNs.

(4) The CMLOA-NLA method is used to detect the (x, and y) coordinates as TN which decreases the localized error. The primitives applied in the localized problem are a mean 4-sided detachment amongst TN as well as AN, as given in Equation (15):

(15) $$f\left(x,y\right)=\frac{1}{N}{\left(\overset{N}{\underset{i=1}{\sum }}\sqrt{{\left(x-x_{i}\right)}^{2}+{\left(y-y_{i}\right)}^{2}}-\hat{d}\right)}^{2}$$

where N≥3 signifies the AN counts are confidential and a transmission radius of TN.

(5) When the maximal repetition counts are acquired, then optimal location coordination (x,y) is defined by the CMLOA-NLA technique.

The localizing error is defined after calculating the localized TN $N_{L}$. It could be assessed as a mean 4-sided of coldness in the node $\left(X_{i},\;Y_{i}\right)$ coordinates in the real node $\left(x_{i},y_{i}\right)$ coordinates, as given in Equation (16).

(16) $$E_{1}=\frac{1}{N_{1}}\overset{N}{\underset{i=1}{\sum }}\sqrt{{\left(x_{i}-X_{i}\right)}^{2}+{\left(y_{i}-Y_{i}\right)}^{2}}.$$

(6) Steps 2–6 are reiterated until the TN can be localized. The localization technique is based on the mistake-controlled $E_{1}$, and the amount of unlocalized bulges $N_{N_{L}}$ is determined as $N_{N_{L}}=M-N_{L}$. The lowest score of $E_{1}$ and $N_{N_{L}}$ shows a restricted approach.

## 4. Results Analysis

The proposed model was simulated using the MATLAB tool. The parameter settings are given as follows. Number of nodes: 100, beacon nodes: 35, communication radius: 35 m, and node deployment: random.

**Figure 3:**
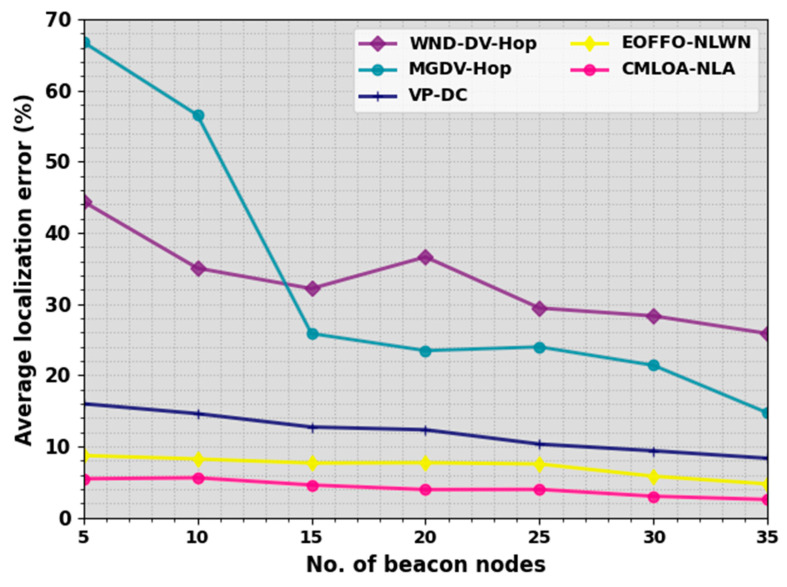
ALE outcome of CMLOA-NLA approach under varying NBNs.

The NL results of the CMLOA-NLA technique are examined in terms of different metrics. In Table 1 and Figure 3, an average localization error (ALE) result of the CMLOA-NLA technique is compared under a distinct number of beacon nodes (NBNs) [28]. ALE measures the accuracy of the node localization. It determines the mean error between the estimated positions and the ground truth positions of the nodes. The results highlight that the CMLOA-NLA technique offers reduced ALE values over all NBN values. On an NBN of 5, the CMLOA-NLA technique provides a decreasing ALE of 5.46% while the WND-DV-Hop, MGDV-Hop, VPDC, and EOFFONLWN models offer an increased ALE of 44.4%, 66.79%, 16%, and 8.73%, respectively. 

The proposed model quantifies that it offers closer estimated positions of nodes to their actual positions. Also, on an NBN of 15, the CMLOA-NLA method gains a lesser ALE of 4.59% while the WND-DV-Hop, MGDV-Hop, VPDC, and EOFFONLWN models offer a bigger ALE of 32.17%, 25.86%, 12.73%, and 7.67%, correspondingly. Furthermore, on an NBN of 35, the CMLOA-NLA system attained a lesser ALE of 2.55% while the WND-DV-Hop, MGDV-Hop, VPDC, and EOFFONLWN approaches achieved a better ALE of 25.86%, 14.71%, 8.34%, and 4.74%, correspondingly.

**Figure 4:**
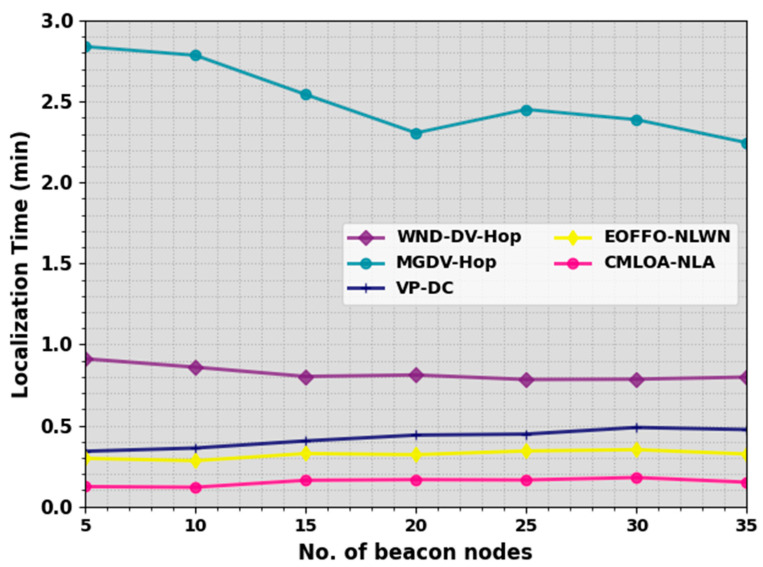
LT outcome of CMLOA-NLA approach under varying NBNs.

In Table 2 and Figure 4, a localization time (LT) study of the CMLOA-NLA algorithm is compared under a distinct NBN. LT represents the time taken by the algorithm to complete the localization process. This is an essential factor, especially in real-time applications where timely and efficient localization is critical. The outcome demonstrates that the CMLOA-NLA system attains lesser LT values over all NBN values. On an NBN of 5, the CMLOA-NLA approach achieves a lesser LT of 0.123 min while the WND-DV-Hop, MGDV-Hop, VPDC, and EOFFONLWN approaches offer an enhanced LT of 0.912 min, 2.839 min, 0.341 min, and 0.297 min, correspondingly. 

Likewise, on an NBN of 15, the CMLOA-NLA technique provides a decreasing LT of 0.162 min while the WND-DV-Hop, MGDV-Hop, VPDC, and EOFFONLWN approaches offer an increased LT of 0.803 min, 2.543 min, 0.405 min, and 0.327 min, correspondingly. Furthermore, on an NBN of 35, the CMLOA-NLA method attains a minimal LT of 0.150 min while the WND-DV-Hop, MGDV-Hop, VPDC, and EOFFONLWN models offer an increased LT of 0.799 min, 2.246 min, 0.475 min, and 0.324 min, correspondingly. The minimal CT values portrayed that the proposed model is suitable for real-time or near real-time applications.

**Figure 5:**
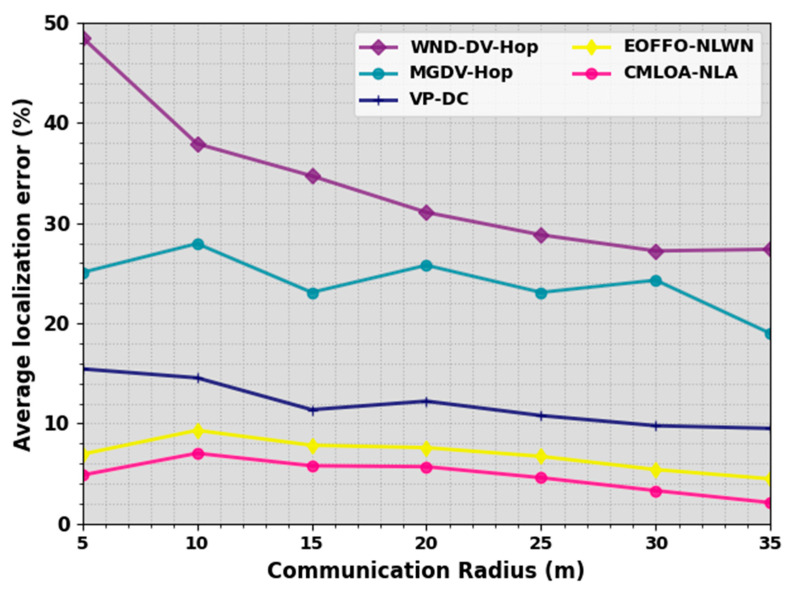
ALE outcome of CMLOA-NLA approach under varying CR.

In Table 3 and Figure 5, an ALE outcome of the CMLOA-NLA methodology is compared on distinct numbers of communication radius (CR). The outcome represented that the CMLOA-NLA algorithm attains lesser ALE values over all CR values. On a CR of 5 m, the CMLOA-NLA technique provides a decreasing ALE of 4.84% while the WND-DV-Hop, MGDV-Hop, VPDC, and EOFFONLWN models offer an increased ALE of 48.53%, 25.07%, 15.44%, and 6.93%, correspondingly. In addition, on a CR of 15 m, the CMLOA-NLA method provides a decreasing ALE of 5.77% while the WND-DV-Hop, MGDV-Hop, VPDC, and EOFFONLWN models offer an enhanced ALE of 34.71%, 23.08%, 11.37%, and 7.82%, correspondingly.

Furthermore, on a CR of 35 m, the CMLOA-NLA technique achieves a lesser ALE of 2.09% while the WND-DV-Hop, MGDV-Hop, VPDC, and EOFFONLWN models offer an increased ALE of 27.38%, 18.98%, 9.50%, and 4.47%, correspondingly.

**Figure 6:**
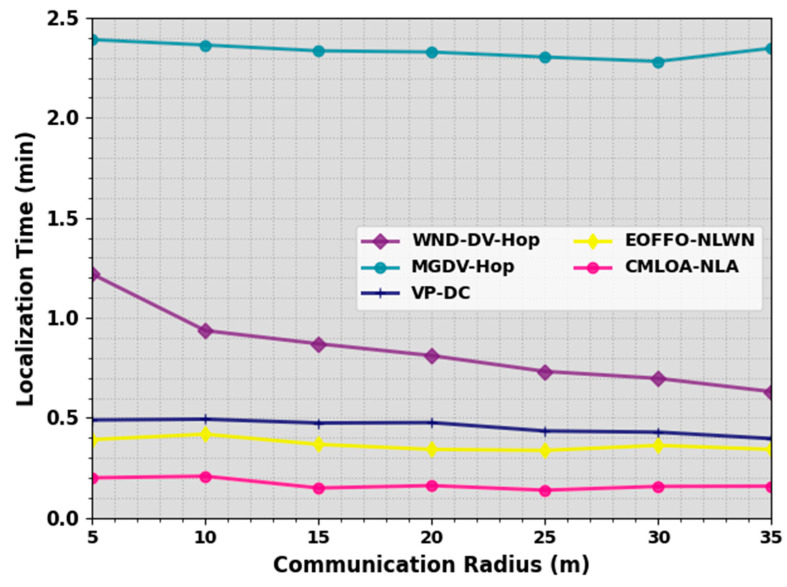
LT outcome of CMLOA-NLA approach under varying CR.

In Table 4 and Figure 6, an LT outcome of the CMLOA-NLA methodology is compared on a distinct number of CRs. The outcome highlights that the CMLOA-NLA algorithm offers reduced LT values over all CR values. On a CR of 5 m, the CMLOA-NLA system provides a lesser LT of 0.200 min while the WND-DV-Hop, MGDV-Hop, VPDC, and EOFFONLWN approaches attain an improved LT of 1.219 min, 2.391 min, 0.489 min, and 0.391 min, correspondingly. Also, on a CR of 15 m, the CMLOA-NLA method offers a lesser LT of 0.149 min while the WND-DV-Hop, MGDV-Hop, VPDC, and EOFFONLWN algorithm offer an enhanced LT of 0.87 min, 2.335 min, 0.474 min, and 0.367 min, correspondingly.

Furthermore, on a CR of 35 m, the CMLOA-NLA methodology provides a lesser LT of 0.158 min while the WND-DV-Hop, MGDV-Hop, VPDC, and EOFFONLWN systems attain a higher LT of 0.631 min, 2.348 min, 0.396 min, and 0.342 min, correspondingly. These results stated the improved NL results of the CMLOA-NLA technique over other models on the WSN.

The presented CMLOA-NLA offers many benefits that make it depict recent localization models. Initially, the CMLOA-NLA approach leverages the model of chaotic mapping that establishes a dynamic and unpredictable element in the optimizer method. This dynamic nature of chaotic mapping can support the method to escape local optima more efficiently, permitting it to converge faster and determine more accurate performances. This is particularly useful in WSNs, but the accuracy of NL is essential for proficient network function and data fusion. By integrating this chaotic mapping model into the standard LOA, the CMLOA-NLA significantly improves the convergence speed and precision of NL. Second, the CMLOA-NLA method concentrates on defining the localization of unknown nodes and relies on ANs that serve as reference points. By optimizing the localization process utilizing LOA, it is optimum to exploit the spatial data provided by the ANs, leading to more particular NL. Widespread simulations and comparative outcomes with existing localization methods show that the CMLOA-NLA system excels in terms of localization accuracy and convergence speed. It is essential in resource-constrained WSNs where energy-efficient and precise NL contributes to enhanced data routing, network lifetime, and overall system performance. In summary, the combination of chaotic mapping and its concentration on AN-based localization in the CMLOA-NLA approach gives its higher performance related to recent approaches, making it a promising outcome for WSN node localization.

## 5. Conclusions

In this study, a new localization approach using a metaheuristic system, named the CMLOA-NLA method for WSN, has been introduced. The purpose of the CMLOA-NLA methodology is to define the localization of UNs based on the ANs as a reference point. In addition, the CMLOA is mainly derived from the combination of the tent chaotic mapping concept with the typical LOA, which tends to improve the convergence speed and precision of NL. With extensive simulations and comparison results with recent localization approaches, the effectual performance of the CMLOA-NLA technique is illustrated. The experimental outcomes demonstrate considerable improvement in terms of accuracy as well as efficiency. Furthermore, the CMLOA-NLA technique was demonstrated to be highly robust against localization errors and transmission ranges. The enhanced accuracy and performance of the CMLOA-NLA technique make it suitable for real-time environmental monitoring, where the accurate localization of nodes is important to assess and respond to environmental changes. Additionally, CMLOA-NLA can be valuable in industrial automation, optimizing data routing in smart factories, and enabling efficient target tracking in surveillance systems. The algorithm’s robustness against localization errors and transmission range variations also positions it as a valuable tool in disaster management scenarios, where reliable and accurate localization of sensors can aid in disaster responses and recovery efforts. Further research can explore its potential in emerging IoT applications, contributing to more effective and reliable sensor network deployments. The CMLOA-NLA is based on the existence of ANs as reference points for NL. In scenarios where ANs are sparse or their situation is suboptimal, this method’s performance can degrade, resulting in less accurate NL. Although this method can show promise in simulation environments, its efficiency and reliability in real-world utilizations could not be thoroughly tested. The transition from simulation to practical execution uncovers unforeseen limitations and problems. This method’s scalability to very large sensor networks can be a concern. As the network size enhances, these methods’ computational demands become expensive, making them less appropriate for large-scale deployments. 

## Figures and Tables

**Figure 2:**
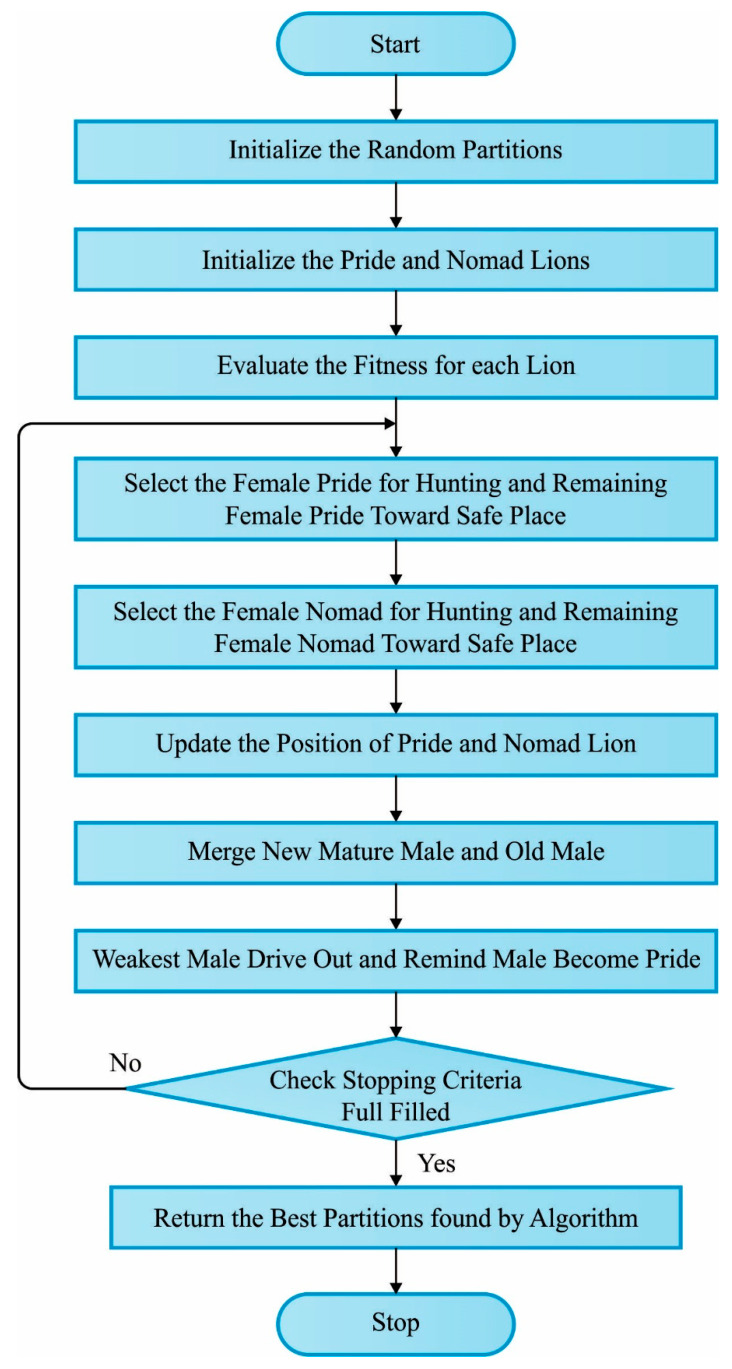
Flowchart of LOA.

**Table 1:** ALE outcome of CMLOA-NLA approach with the recent system under varying NBNs.

Average Localization Error (%)					
No. of Beacon Nodes	WND-DV-Hop	MGDV-Hop	VPDC	EOFFONLWN	CMLOA-NLA
5	44.40	66.79	16.00	8.73	5.46
10	35.04	56.54	14.61	8.25	5.60
15	32.17	25.86	12.73	7.67	4.59
20	36.62	23.46	12.34	7.72	3.95
25	29.44	23.98	10.33	7.54	3.97
30	28.33	21.39	9.39	5.82	3.01
35	25.86	14.71	8.34	4.74	2.55

**Table 2:** LT outcome of CMLOA-NLA approach with the recent system under varying NBNs.

Localization Time (min)					
No. of Beacon Nodes	WND-DV-Hop	MGDV-Hop	VPDC	EOFFONLWN	CMLOA-NLA
5	0.912	2.839	0.341	0.297	0.123
10	0.860	2.785	0.361	0.283	0.119
15	0.803	2.543	0.405	0.327	0.162
20	0.812	2.306	0.441	0.320	0.166
25	0.784	2.451	0.447	0.343	0.164
30	0.786	2.388	0.488	0.351	0.179
35	0.799	2.246	0.475	0.324	0.150

**Table 3:** ALE outcome of CMLOA-NLA approach with the recent system on distinct CR.

Average Localization Error (%)					
Communication Radius (m)	WND-DV-Hop	MGDV-Hop	VPDC	EOFFONLWN	CMLOA-NLA
5	48.53	25.07	15.44	6.93	4.84
10	37.92	27.95	14.55	9.31	7.02
15	34.71	23.08	11.37	7.82	5.77
20	31.07	25.79	12.22	7.58	5.68
25	28.82	23.08	10.78	6.71	4.58
30	27.22	24.30	9.77	5.39	3.29
35	27.38	18.98	9.50	4.47	2.09

**Table 4:** LT outcome of CMLOA-NLA approach with the recent system under varying CR.

Localization Time (min)					
Communication Radius (m)	WND-DV-Hop	MGDV-Hop	VPDC	EOFFONLWN	CMLOA-NLA
5	1.219	2.391	0.489	0.391	0.200
10	0.936	2.364	0.493	0.418	0.208
15	0.87	2.335	0.474	0.367	0.149
20	0.811	2.329	0.476	0.342	0.161
25	0.732	2.304	0.434	0.337	0.138
30	0.697	2.282	0.428	0.362	0.157
35	0.631	2.348	0.396	0.342	0.158

## Data Availability

Data sharing does not apply to this article as no datasets were generated during the current study.
